# Binding of smoothelin-like 1 to tropomyosin and calmodulin is mutually exclusive and regulated by phosphorylation

**DOI:** 10.1186/s12858-017-0080-6

**Published:** 2017-03-21

**Authors:** Annegret Ulke-Lemée, David Hao Sun, Hiroaki Ishida, Hans J. Vogel, Justin A. MacDonald

**Affiliations:** 1 Department of Biochemistry & Molecular Biology, University of Calgary, Cumming School of Medicine, 3280 Hospital Drive NW, Calgary, AB T2N 4Z6 Canada; 2 Biochemistry Research Group, Department of Biological Sciences, University of Calgary, 2500 University Drive NW, Calgary, AB T2N 1 N4 Canada

**Keywords:** SMTNL1, CHASM, Smooth muscle, Thin filament, Protein binding

## Abstract

**Background:**

The smoothelin-like 1 protein (SMTNL1) can associate with tropomyosin (Tpm) and calmodulin (CaM), two proteins essential to the smooth muscle contractile process. SMTNL1 is phosphorylated at Ser301 by protein kinase A during calcium desensitization in smooth muscle, yet the effect of SMTNL1 phosphorylation on Tpm- and CaM-binding has yet to be investigated.

**Results:**

Using pull down studies with Tpm-Sepharose and CaM-Sepharose, we examined the interplay between Tpm binding, CaM binding, phosphorylation of SMTNL1 and calcium concentration. Phosphorylation greatly enhanced the ability of SMTNL1 to associate with Tpm in vitro; surface plasmon resonance yielded a 10-fold enhancement in *K*_D_ value with phosphorylation. The effect on CaM binding is more complex and varies with the availability of calcium.

**Conclusions:**

Combining both CaM and Tpm with SMTNL1 shows that the binding to both is mutually exclusive.

**Electronic supplementary material:**

The online version of this article (doi:10.1186/s12858-017-0080-6) contains supplementary material, which is available to authorized users.

## Background

Smoothelin-like 1 protein (SMTNL1), also termed calponin homology-associated with smooth muscle (CHASM) protein, was originally identified as a protein phosphorylated in response to cyclic nucleotide-dependent protein kinase (i.e., protein kinase A (PKA) and protein kinase G (PKG)) activation during calcium desensitization of gastrointestinal smooth muscle [1]. Additional studies have revealed a role for SMTNL1 in vascular smooth muscle contractile activity, as well as cardiovascular and skeletal muscle adaptation to exercise, development and pregnancy [2–6].

The ubiquitous calmodulin (CaM) is the primary cellular signal transducer that provides spatial and temporal responses to changes in intracellular calcium levels [7]. CaM provides signaling by association with specific binding sites in target proteins, both in its calcium-saturated (Ca-CaM) or its calcium-free (apo-CaM) form [8, 9]. We have previously reported that SMTNL1 possesses two CaM-binding sites: a classic CaM-binding domain (CBD1, amino acids 310–325) in the center of the protein as well as an IQ-motif that serves as an apo-CaM-binding site (CDB2, amino acids 439–457) located in the carboxy-terminal calponin homology domain [10–12]. SMTNL1 was established as a *bona fide* CaM-binding protein within aortic A7r5 smooth muscle cells using the proximity ligation assay. In this regard, CBD1 is thought to provide the majority of CaM-binding in situ; however, CBD2 may contribute cooperatively to binding [12].

A second binding partner of SMTNL1 has also been identified in smooth muscle. Tropomyosin (Tpm), a muscle protein that aids in the stabilization of actin filaments, is incorporated as super-helical polymers along smooth muscle thin filaments and contribute to the regulation of actin-myosin cross bridge cycling during smooth muscle contraction [13–15]. In smooth muscle, two Tpm isoforms (Tpm1.4 and Tpm2.1; previously identified as Tmsm-α/Tm6 and Tmsm-β/Tm1β, respectively [16]) are predominantly expressed [17]. SMTNL1 was shown to associate with smooth muscle Tpm α/β dimers with a binding surface comprising of its N-terminal intrinsically-disordered region and the C-terminal calponin homology domain [18].

Previous in vitro studies have defined SMTNL1 as a Tpm- and CaM-binding protein [10–12, 18, 19]. Moreover, the phosphorylation of SMTNL1 was identified during calcium desensitization of smooth muscle [1, 5]. However, the effect of phosphorylation on the ability of SMTNL1 to associate with Tpm and/or CaM has not been investigated. Intriguingly, the binding domains for CaM (CBD1 and CBD2) are localized in close vicinity to the Tpm-binding surface on the SMTNL1 protein. In addition, the Ser301 phosphorylation site targeted by cyclic nucleotide-dependent protein kinases (i.e., PKA and PKG) is located near CBD1 and within the Tpm-binding region [11, 18]. This led us to inquire about the interplay of SMTNL1 phosphorylation and calcium levels on the multi-functionality of Tpm- and CaM-binding. Herein, we demonstrate that phosphorylation of Ser301 greatly enhanced the ability of SMTNL1 to associate with smooth muscle Tpm in vitro. The effect of Ser301 phosphorylation on CaM binding was more complex and varied with the availability of calcium. Combining both CaM and Tpm revealed that binding with SMTNL1 was mutually exclusive.

## Methods

### Materials

PreScission Protease, glutathione-Sepharose and CNBr-activated Sepharose were from GE Healthcare (Piscataway, NJ). The Phos-tag acrylamide reagent was from NARD Chemicals (Kobe City, Japan). Smooth muscle Tpm was a purified α/β heterodimer of chicken gizzard Tpm1.4_sm(a.a.b.d)_ (equivalent to Tm6 or Tmsm-α) and Tpm2.1_sm(a.b.a.d)_ (equivalent to TM-1, βTm or Tmsm-β) [16] and was provided by Dr. Michael Walsh (University of Calgary). CaM was provided by Dr. Hans Vogel (University of Calgary). PKA was purified from bovine heart using the method described in [20].

### Expression and Purification of SMTNL1

A clone comprising the Tpm-binding region of SMTNL1 (SMTNL1-TMB, amino acids 195–459; NP_077192) was described previously [18, 19]. SMTNL1-TMB was expressed as a glutathione *S*-transferase (GST)-fusion protein in *E. coli* and purified with glutathione-Sepharose. The fusion protein was cleaved with PreScission Protease, and GST was removed by an additional pass over glutathione-Sepharose along with a final clean-up using MonoQ anion exchange chromatography. In some cases, SMTNL1-TMB was phosphorylated with PKA at a molar ratio of 500:1 (SMTNL1: PKA) in a buffer consisting of 2 mM MgCl_2_, 0.2 mM ATP, 25 mM HEPES and 150 mM NaCl. The phosphorylated protein was buffer-exchanged and concentrated using a 3 kDa cut-off centrifugation filter unit (EMD Millipore, Billerica, MA).

### Tpm-Sepharose binding assay

Purified smooth muscle Tpm or Tris-HCl (as control) was covalently bound to CNBr-activated Sepharose following the manufacturers’ instructions and as described in [19]. SMTNL1-TMB (200 μg) was incubated with 40 μL of Tpm-Sepharose or Control-Sepharose in binding buffer (20 mM Tris-HCl, pH 7.5 with 0.1% (v/v) β-mercaptoethanol) for 2 h at 4 °C and then washed extensively with the same buffer supplemented with 150 mM NaCl. Bound protein was eluted with SDS-PAGE loading buffer, separated by gel electrophoresis, and detected by Coomassie stain.

### Calmodulin-Sepharose binding assay

Calmodulin (CaM)-Sepharose pull-down experiments were completed as described previously [11]. Briefly, SMTNL1-TMB (200 μg) was incubated with 40 μL of CaM-Sepharose in binding buffer (20 mM HEPES, pH 7.0 in the presence of 5 mM CaCl_2_ (Ca-CaM) or 1 mM EDTA (apo-CaM)). After incubation for 1 h at 4 °C, the CaM-Sepharose was washed extensively with the same buffer supplemented with 150 mM NaCl. Bound SMTNL1-TMB protein was eluted with SDS-PAGE loading buffer, separated by gel electrophoresis, and detected by Coomassie stain. In some experiments, SMTNL1-TMB was premixed with Tpm in different molar ratios, allowed to form complexes for 1 h at 4 °C and then added to the CaM-Sepharose.

### Surface Plasmon Resonance (SPR)

The binding between Tpm and SMTNL1-TMB with or without phosphorylation was evaluated by SPR using a BIAcore X100 instrument (GE Healthcare). Purified Tpm was immobilized via amine-coupling onto a CM5 sensor chip (GE Healthcare). The running buffer contained 20 mM HEPES pH 7.5, 100 mM KCl, 1 mM DTT, and 0.005% (v/v) Tween-20. Five concentrations of SMTNL1-TMB from 0.12 to 10 μM were prepared by serial dilution and were injected at a flow rate of 30 μL/min with a contact time of 1 min at 25 °C. The chip was regenerated by injecting 1 M NaCl for 4 min followed by glycine-HCl (10 mM, pH 3.0) for 1 min. Each experiment was repeated three times (*n* = 3) to obtain a standard error (SE). The BIAevaluation software 2.0 (GE Healthcare) was used to analyze the SPR sensorgrams and to obtain the dissociation constants (Kd).

### Data analysis

Values are presented as the mean ± S.E.M., with *n* indicating the number of independent experiments. Data were analyzed with two-tailed Student’s *t* test, or for comparison of multiple groups, with one-way analysis of variance (ANOVA) and Tukey’s *post hoc* test. Differences were considered to be statistically significant for *p* < 0.05. All statistical analyses were performed using the GraphPad Prism 6.0 program.

## Results

### Phosphorylation of SMTNL1 by PKA enhances Tpm-binding potential

**Fig. 1:**
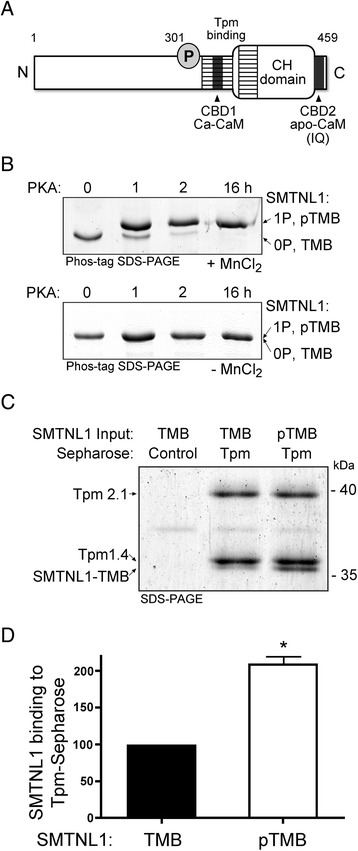
The phosphorylation of SMTNL1 by PKA alters its tropomyosin-binding potential. **a** The SMTNL1 protein contains: a *C*-terminal calponin homology (CH) domain, Ser301 PKA-phosphorylation site, Ca-calmodulin (CaM)-binding domain (CBD1), apo-CaM-binding domain (CBD2), and tropomyosin (Tpm)-binding domain (indicated as the hatched area). **b** Purified recombinant SMTNL1-TMB was incubated with PKA as described in the Methods section. Samples were withdrawn at the indicated times and subjected to Phos-tag SDS-PAGE; two discrete bands representing unphosphorylated SMTNL1-TMB (0P, TMB) or SMTNL1-TMB phosphorylated at Ser301 (1P, pTMB) were detected by Coomassie stain. Samples subjected to Phos-tag SDS-PAGE in the absence of MnCl_2_ confirmed the shift in band migration to be a result of phosphorylation. **c** Unphosphorylated or phosphorylated SMTNL1-TMB (200 μg) was incubated with 40 μL of Tpm-Sepharose (α/β-heterodimer: Tpm1.4/Tpm2.1). Bound SMTNL1-TMB was eluted with boiling 0.1% SDS solution and detected with Coomassie staining of SDS-PAGE gels. **d** The SMTNL1-TMB bands were quantified by densitometry, and binding to Tpm-Sepharose was expressed as percentage of the SMTNL1-TMB binding found for the unphosphorylated state. All experiments are *n* = 3–5 and were analyzed by Student’s *t*-test. *- Significantly different from unphosphorylated SMTNL1-TMB, *p* < 0.05

Full-length SMTNL1 protein exhibits reduced stability that complicates conclusions from in vitro binding experiments. A truncated form of SMTNL1 (i.e., SMTNL1-TMB) was used since this clone is stable under the conditions and times used; furthermore, SMTNL1-TMB contains all known functional elements of SMTNL1 (e.g., CBD1, CBD2, calponin-homology (CH) domain, Tpm-binding regions and Ser301 phosphorylation site; Fig. 1a). SMTNL1-TMB could be effectively phosphorylated with PKA; the phosphorylation stoichiometry was assessed with Phos-tag SDS-PAGE and judged to be complete (i.e., ~ 1 mol phosphate/mol SMTNL1) after 16 h incubation at 4 °C (Fig. 1b). The binding of SMTNL1-TMB to Tpm was examined in pull-down assays with purified smooth muscle Tpm (mixture of Tpm1.4 and Tpm2.1 isoforms) immobilized to Sepharose resin (Tpm-Sepharose). SMTNL1-TMB was recovered from Tpm-Sepharose while no interaction with Control-Sepharose was observed (Fig. 1c). Some Tpm could be released from the Sepharose column with application of SDS elution buffer and boiling. This was anticipated given that smooth muscle Tpm exists as coiled-coil heterodimers [21], and it was unlikely that all Tpm molecules could be covalently coupled to the CNBr-Sepharose resin. Approximately 2-fold more phosphorylated SMTNL1-TMB could be recovered from pull-down assays with Tpm-Sepharose (Fig. 1d) when compared with unphosphorylated SMTNL1-TMB. Binding constants for a kinetic series, 0–10 μM phosphorylated SMTNL1-TMB provided a *K*_D_ of 3.2 × 10^−7^M with biophysical analyses by SPR (Additional file 1: Figure S1), a response that was enhanced 10-fold when compared to unphosphorylated SMTNL1-TMB (3.0 × 10^−6^M). The SPR assays provided a similar *K*_D_ for unphosphorylated SMTNL1 and Tpm as previously defined by isothermal titration calorimetry (ITC) assays [19]. We previously reported that the SMTNL1 binding surface for Tpm included a central portion of the intrinsically-disordered region, located proximal to the Ser301 phosphorylation site, as well as a surface of the calponin homology (CH) domain located at the *C*-terminus [18]. So, it is not unexpected that phosphorylation at Ser301 could alter the electrostatic properties and/or the intramolecular conformation of the Tpm-binding surface on SMTNL1.

### Phosphorylation of SMTNL1-TMB influences binding to CaM-Sepharose

**Fig. 2:**
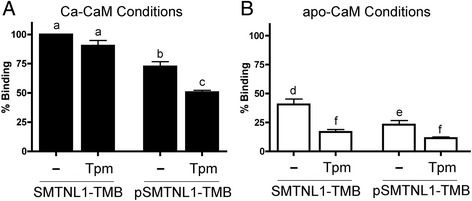
Interdependency of SMTNL1 phosphorylation, calcium and tropomyosin on calmodulin-binding. SMTNL1-TMB (200 μg, 7 nmol) was incubated with an equimolar amount of Tpm. The mixture was then incubated with CaM-Sepharose (40 μL; ligand density of ~10–14 μmol/mL) in the presence (**a**; Ca-CaM, 5 mM CaCl_2_) or absence (**b**; apo-CaM, 1 mM EDTA) of calcium. Some experiments were completed with SMTNL1-TMB that had been previously phosphorylated with PKA. After washing, the retention of SMTNL1-TMB or phosphorylated SMTNL1-TMB was analyzed. The band densities were quantified and binding to CaM-Sepharose expressed as percentage of SMTNL1-TMB recovered under maximal binding conditions (i.e., Ca-CaM in the absence of Tpm). All experiments are *n* = 3–5 and were analyzed by one-way ANOVA with Tukey’s *post hoc* analysis. Different letters indicate significant differences among groups (a,b,c: comparison among all Ca-CaM conditions; d,e,f: comparison among all apo-CaM conditions; *p* < 0.05)

The Ser301 phosphorylation site of SMTNL1 is located in close proximity to the Ca-CaM binding domain (CBD1, amino acids 310–325 [11]). It was expected that the phosphorylation of SMTNL1-TMB by PKA would impact the association with Ca-CaM and not with apo-CaM, since the binding of the latter was linked to an IQ-domain at the C-terminus (CBD2, amino acids 439–457 [10]). Indeed, we observed reductions in Ca-CaM-Sepharose binding for phosphorylated SMTNL1-TMB, with recovery of approximately 30% less bound material (Fig. 2a). Surprisingly, the phosphorylation of SMTNL1-TMB protein also impacted upon binding to apo-CaM-Sepharose (Fig. 2b). Although a general reduction in SMTNL1-TMB bound to CaM was observed in the apo condition (i.e., ~60% less SMTNL1-TMB recovered on the resin), the recovery of phosphorylated SMTNL1-TMB from the resin was further reduced to approximately 20% of the unphosphorylated material (Fig. 2b). We have previously demonstrated that the binding of SMTNL1-TMB to CaM was influenced by calcium, with SMTNL1 binding to CaM-Sepharose decreased by approximately 50% in the apo-calcium condition [11].

### Phosphorylation and calcium influence SMTNL1-TMB binding to CaM-Sepharose in the presence of Tpm

Equimolar amounts of SMTNL1-TMB and Tpm proteins were pre-incubated and then applied to CaM-Sepharose so that competition between CaM and Tpm for binding to SMTNL1-TMB could be assessed. Binding of SMTNL1-TMB to apo-CaM-Sepharose was reduced by approximately 60% by the addition of Tpm to SMTNL1-TMB prior to capture with the resin (Fig. 2b). These data indicate that Tpm could interfere with the apo-CaM binding properties of SMTNL1-TMB, likely due to the proximity of the two binding sites within the calponin homology (CH) domain. Furthermore, we can conclude that SMTNL1-TMB interacts with Tpm more strongly than with apo-CaM. This was not unexpected as the interaction of SMTNL1 with apo-CaM is weak (Kd ~10^−6^ M [10]). The results of the binding assays were not affected by the order of addition of proteins since pre-incubating SMTNL1-TMB with CaM-Sepharose followed by competition with Tpm showed the same reduction in binding (data not shown). The subsequent addition of Tpm to the mixture of phosphorylated SMTNL1 and apo-CaM-Sepharose, shown in Fig. 2b, further decreased binding potential to barely detectably levels. When investigating interactions with Ca-CaM, the binding of unphosphorylated SMTNL1-TMB to Ca-CaM-Sepharose was not significantly influenced by the addition of equimolar amounts of Tpm (Fig. 2a). However, the addition of Tpm did significantly reduce the recovery of phosphorylated SMTNL-TMB on the Ca-CaM-Sepharose resin.

### SMTNL1 phosphorylation diminishes Ca-CaM-binding potential under conditions of high Tpm content

**Fig. 3:**
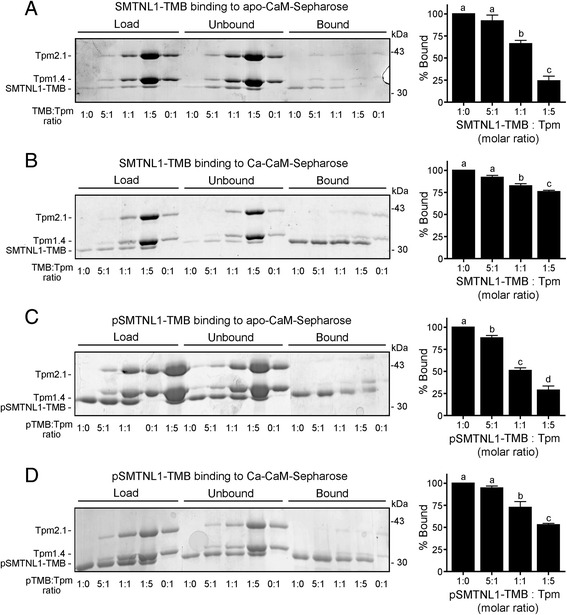
SMTNL1 phosphorylation precludes Ca-CaM-binding in favour of Tpm-binding under conditions of high Tpm content. SMTNL1-TMB or phosphorylated SMTNL1-TMB (concentrations as described in Fig. 2) was pre-incubated with the indicated molar ratio of purified α/β Tpm dimer. The mixtures were then added to CaM-Sepharose in the absence (**a**, **c**; apo-CaM, 1 mM EDTA), or presence (**b**, **d**; Ca-CaM, 5 mM CaCl_2_) of calcium. After extensive washing, bound SMTNL1-TMB was eluted and detected with Coomassie staining of SDS-PAGE gels. The band density was quantified and binding expressed as percentage of maximum binding within each condition. All experiments are *n* = 3 and were analyzed by one-way ANOVA with Tukey’s *post hoc* analysis. Different letters indicate significant differences among groups (*p* < 0.05)

The intracellular Tpm concentration is predicted to be significantly higher than that of SMTNL1, as judged from the relative staining intensities of 2D-SDS-PAGE gels [1]. So, the incubation of SMTNL1-TMB with different molar ratios of Tpm prior to the addition of CaM-Sepharose was used to reveal additional information about the character of the protein complex formation. Increasing the amounts of Tpm resulted in a notable decrease in the binding of SMTNL1-TMB to apo-CaM-Sepharose (Fig. 3a). Moreover, there was minimal influence of increased Tpm on the binding of phosphorylated SMTNL1-TMB in the apo-CaM condition (Fig. 3b). The small inhibitory effect of increasing Tpm concentration on the amount of SMTNL1-TMB retained on Ca-CaM Sepharose suggests a greater affinity of SMTNL1-TMB for Ca-CaM over Tpm (Fig. 3c). However, distinct effects were observed for Ca-CaM binding of phosphorylated SMTNL1-TMB under conditions of high Tpm (Fig. 3d). In this case, the phosphorylation of SMTNL1-TMB suppressed Ca-CaM binding potential in favour of Tpm.

## Discussion

Phosphorylation events are known to regulate binding of CaM to target proteins. Several examples of phosphorylation within or near CaM-binding domains (CBDs) have been reported in the literature, including but not limited to smooth muscle myosin light chain kinase [22], MARKS protein [23], plasma membrane Ca^2+^-ATPase [24], endothelial nitric oxide synthase (eNOS) [25], and the Ca^2+^-dependent K^+^-channel [26]. CaM binds to its targets by wrapping around a short amphipathic helix within the CBD [8, 9], thus the phosphorylation of residues within a CBD can disrupt CaM interactions even if the Ser/Thr residues subject to phosphorylation are not necessarily required for CaM binding [22]. Herein, we describe a novel incidence where phosphorylation regulates CaM binding to a target protein. In this case, the PKA-dependent phosphorylation of SMTNL1 at Ser301 modulates both Ca- and apo-CaM binding. Ser301 lies well upstream of the core hydrophobic residues within CBD1 required for Ca-CaM-binding (i.e., 20 amino acids from Phe321 which is the critical contributor [11]). It is likely that the distance between Ser301 and the hydrophobic patch of CBD1 is too distant for phosphorylation to directly block Ca-CaM docking. This agrees with our observation that the rate of PKA-dependent phosphorylation at Ser301 was not influenced when apo- or Ca-CaM was included in the kinase assay (unpublished results). We have previously demonstrated that intramolecular interactions between the *C*-terminal IQ motif (localization of the apo-CaM binding site, CBD2) and the intrinsically disordered region (localization of the phosphorylation site) influence apo-CaM binding [11, 12]. Furthermore, a portion of the *N*-terminal intrinsically disordered region forms intramolecular contacts with the globular *C*-terminal calponin homology (CH) domain [18]. Thus, it is conceivable that phosphorylation within the *N*-terminal region has far-reaching effects in the *C*-terminal domain and can attenuate both Ca- and apo-CaM binding potentials.

The phosphorylation of SMTNL1-TMB enhanced the Tpm-binding potential in both pull down studies and by confirming SPR assays. Inconsequential amounts of nonspecific Tpm-binding were found with CaM-Sepharose, and its binding was not influenced by the presence of SMTNL1-TMB, irrespective of phosphorylation or calcium. Thus, we conclude that a heterotrimeric complex of SMTNL1-TMB, CaM and Tpm was not formed under any of the binding conditions employed. Since no heterotrimer was generated, we suggest that Tpm and Ca-CaM compete for a similar binding site on SMTNL1-TMB. This conclusion is further supported by the fact that the binding surfaces for CaM and Tpm overlap on SMTNL1. Indeed, only very weak binding of Tpm is observed with the isolated CH domain (containing: CBD2; apo-CaM binding) in the absence of the intrinsically-disordered upstream sequence (containing: CBD1, Ca-CaM binding; and Ser301, phosphorylation consensus sequence) [18].

Our findings place SMTNL1 in a position to influence both Ca^2+^/CaM-regulated contractile events and Ca^2+^ desensitization pathways in smooth muscle. Depending on intracellular calcium concentration and cyclic nucleotide-dependent kinase activation, SMTNL1 is predicted to cycle between Tpm-bound and Ca-CaM-bound complexes. The impact of SMTNL1 on smooth muscle contractile processes may be via its binding partners and associations with the contractile filament. In its dephosphorylated state SMTNL1 has weaker affinity for Tpm and would be more likely to associate with CaM. With an increase in intracellular calcium due to a contractile signal, Ca-CaM could bind to SMTNL1 and initiate the dissociation of the complex from the thin filament. Unphosphorylated SMTNL1 can inhibit myosin light chain phosphatase (MLCP) activity in vitro [27], so the release of SMTNL1 could attenuate MLCP-dependent dephosphorylation of myosin regulatory light chain (LC20). Conversely, SMTNL1 phosphorylation during calcium desensitization of smooth muscle could provide enhanced binding to Tpm and restrict binding of CaM in the apo-state, securing phosphorylated SMTNL1 with Tpm on the thin filament. The weak binding modes of SMTNL1 with apo-CaM might not possess enough stability to occur in vivo [12]. While the physiological significance of SMTNL1 association with the contractile filament in situ has not been investigated, we speculate that the protein may act in analogous manners to caldesmon or calponin on actin-myosin cross bridge cycling. Calponin interacts with many cytoskeleton and related proteins (including tropomyosin) and functions as an inhibitor of actin-activated myosin ATPase [28]. The binding of Ca-CaM dissociates calponin from the actin filament to facilitate smooth muscle contraction [29]. Calponin can also be phosphorylated in response to cyclic nucleotide signals to cause a reduced affinity for the thin filament [30]. Caldesmon is another actin filament-associated regulatory protein; it also binds to Tpm and regulates cross-bridge cycling in a Ca-CaM dependent manner [31]. A detailed physiological analysis of the CaM- and Tpm-interactions of SMTNL1 and their functional implications are required to provide a clear description of role of SMTNL1 in smooth muscle contractility. Further investigation will be needed to verify the role of SMTNL1 in fine-tuning thin filament dynamics.

## Conclusions

Herein, we provide evidence that calcium availability increases CaM association with SMTNL1, and CaM and tropomyosin binding to SMTNL1 are mutually exclusive events. Moreover, SMTNL1 binding to CaM is reduced by phosphorylation of Ser301 while binding to tropomyosin is enhanced. We propose a model whereby SMTNL1 can exist in distinct complexes with either Tpm or CaM, depending on availability of calcium and activity of PKA. This switching mechanism could aid in the fine-tuning of smooth muscle contraction.

## Additional file

Additional file 1: Figure S1. SPR measurements for binding of SMTNL1-TMB to tropomyosin. Sensorgrams and data fittings are provided for unphosphorylated and phosphorylated (S301 with PKA) SMTNL1-TMB protein with tropomyosin immobilized via amine-coupling to a CM5 sensor chip. (DOCX 714 kb)

## Abbreviations

apo-CaM: Calcium-free calmodulin
Ca-CaM: Calcium-saturated calmodulin
CaM: Calmodulin
CBD1 and CBD2: Calmodulin binding domain 1 and 2, respectively
PKA: Protein kinase A
PKG: Protein kinase G
SMTNL1: Smoothelin-like 1
SMTNL1-TMB: Tropomyosin-binding region of SMTNL1
Tpm: Tropomyosin
