# Supplementary material for: Binding of smoothelin-like 1 to tropomyosin and calmodulin is mutually exclusive and regulated by phosphorylation
Source: BMC Biochem. 2017 Mar 21;18:5. doi: 10.1186/s12858-017-0080-6 (PMC5359911; doi:10.1186/s12858-017-0080-6)

**Additional file 1: Figure S1.** SPR measurements for binding of SMTNL1-TMB (**A**, phosphorylated with PKA; **B**, unphosphorylated) to tropomyosin immobilized via amine-coupling onto a CM5 sensor chip. Sensorgrams and fittings (1:1 binding model) are shown in solid grey lines and dashed black lines, respectively. The concentration of protein sample injected is also indicated within each sensorgram.

**A**

 
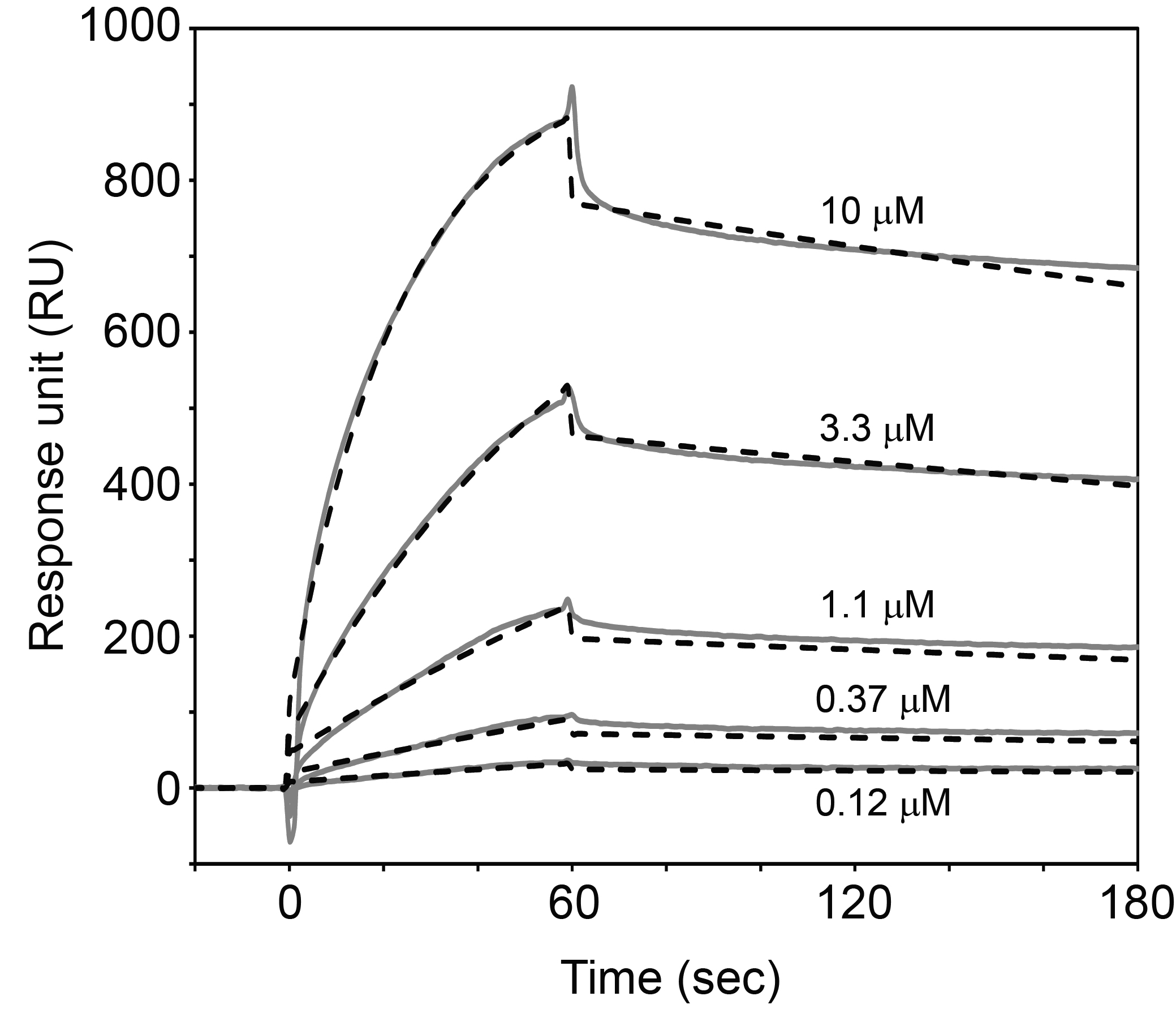


**B**


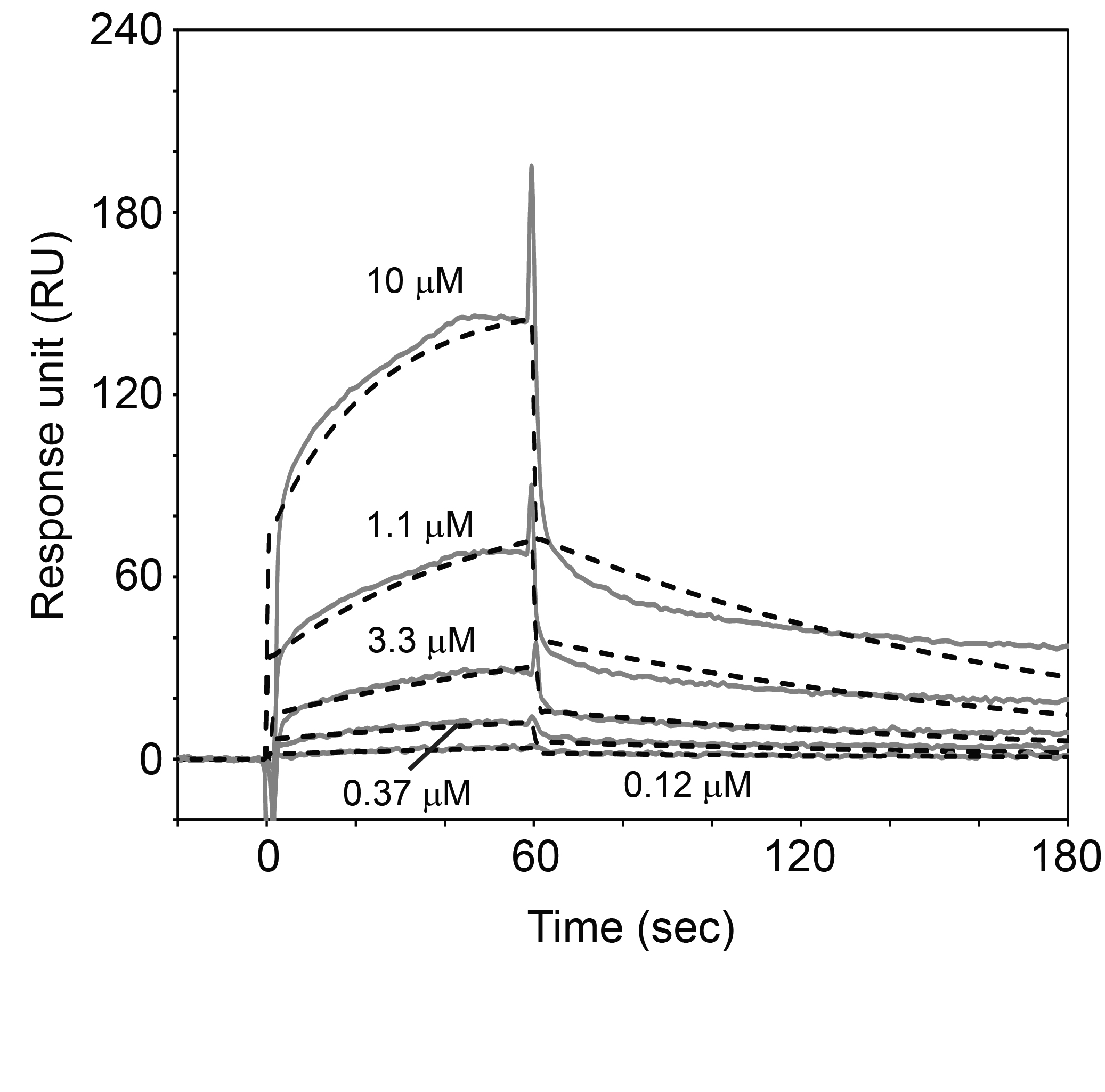

Supplement: Additional file 1: Figure S1. — SPR measurements for binding of SMTNL1-TMB to tropomyosin. Sensorgrams and data fittings are provided for unphosphorylated and phosphorylated (S301 with PKA) SMTNL1-TMB protein with tropomyosin immobilized via amine-coupling to a CM5 sensor chip. (DOCX 714 kb) [file 12858_2017_80_MOESM1_ESM.docx]
